# Supplementary material for: Admission High-Sensitive Cardiac Troponin T Level Increase Is Independently Associated with Higher Mortality in Critically Ill Patients with COVID-19: A Multicenter Study
Source: J Clin Med. 2021 Apr 13;10(8):1656. doi: 10.3390/jcm10081656 (PMC8070238; doi:10.3390/jcm10081656)
Supplement: Supplementary file 1 [file jcm-10-01656-s001.pdf]

Supplementary Materials

Table 1. Echocardiographic evaluation in 66 patients with critical COVID-19.

	Total (n = 66)	Normal Cardiac Function (n = 51)	Cardiac Dysfunction (n = 15)	p-Value
LVEF [1] (%), median [IQR]	60 [50–60]	60 [55–60]	45 [37.5-50]	-
TAPSE [2] (mm), median [IQR]	18 [16–22]	19 [17–22]	13.5 [12-20]	0.02
hs-cTnT [3] (ng/L), median [IQR]	14.7 [9.7–30.0]	13.4 [8.2–29.8]	17.9 [13.4-56.9]	0.12
Death, n (%)	18 (27.3%)	13 (25.5%)	5 (33.3%)	0.55

[1] LVEF: left ventricular ejection fraction, [2] TAPSE: tricuspid annular plane systolic excursion, [3] hs-cTnT: high-sensitive cardiac troponin T.

Table 2. Risk factors associated with the need for mechanical ventilation in critically ill COVID-19 patients.

	Univariate Analysis Hazard Ratio (IC 95%)	p-Value	Multivariable Analysis Hazard Ratio (IC 95%)	p-Value
Age	1 (1–1)	0.04	1.01 (0.98–1.05)	0.47
Male sex	0.97 (0.58–1.6)	0.89		
BMI [1]	1 (0.99–1.1)	0.17	1.02 (0.98–1.07)	0.25
Smokers	1.6 (0.57–4.4)	0.38		
Charlson index	3.2 (1.2–8.6)	0.02	0.68 (0.11–4.14)	0.68
SAPS II [2]	8.4 (2.4–29)	<0.001	5.20 (1.24–21.83)	0.02
PaO_2/FiO_2	1 (0.99–1)	0.01	1.003 (0.99–1)	0.08
Norepinephrine (>1µg/kg/min)	1.9 (1.2–3)	0.007	1.47 (0.90–2.40)	0.12
Renal replacement therapy	1.2 (0.68–2.2)	0.51		
Creatininemia	1.3 (0.46–3.4)	0.66		
Hs-cTnT [3]	1.9 (1.1–3.3)	0.02	1.51 (0.71–3.19)	0.29
CRP [4]	1.8 (0.8–4.1)	0.16	1.36 (0.61–3.02)	0.45
PCT [5]	1.1 (0.75–1.7)	0.59		
Lactatemia	2 (0.4–9.7)	0.41		
D-dimer	1.7 (0.76–3.6)	0.20		

[1] BMI: Body Mass Index, [2] SAPS II: Simplified Acute Physiology Score II, [3] hs-cTnT: high-sensitive cardiac troponin T, [4] CRP: C reactive protein, [5] PCT: procalcitonin
